# Use of Complementary Alternative Medicine and the Associated Factors among Patients with Depression

**DOI:** 10.1155/2021/6626394

**Published:** 2021-03-26

**Authors:** Hamide Ashraf, Alireza Salehi, Malihe Sousani, Mohammad Hossein Sharifi

**Affiliations:** ^1^Department of Persian Medicine, School of Medicine, Shiraz University of Medical Sciences, Shiraz, Iran; ^2^Research Center for Traditional Medicine and History of Medicine, Shiraz University of Medical Sciences, Shiraz, Iran

## Abstract

Complementary Alternative Medicine (CAM) has been widely used in the world, but limited data are available on the use of CAM in depression. This study aimed to evaluate the use of CAM and its associated factors in depression. This cross-sectional study was conducted on 300 depressed patients referred to the Yasuj Neurology and Psychiatric Clinic, southern Iran, between 2019 and 2020. A valid semistructured international questionnaire was used; amongst the participants, 230 (77%) were female. The mean age of the patients was 41.47 ± 12.2 years and the mean duration of the disease was 4.49 ± 4.88 years. The prevalence of CAM use was 37.6% among the patients. The results showed a significant difference between the CAM users and nonusers regarding the disease duration (*p*=0.045) and body mass index (*p*=0.007). Moreover, the results of logistic regression analysis revealed a significant relationship between CAM use and female gender, disease duration, overweight, obesity, and self-employment (*p*=0.039, *p*=0.028, *p*=0.029, *p*=0.048, and *p*=0.044, resp.). The most frequently used type of CAM was herbal medicine (97.35%) followed by pray therapy (23.89%). Additionally, the most widely used herbs were borage (77%), chamomile (46.9%), and lavender (21.2%). Furthermore, 62.8% of the patients reported that their main reason for using CAM was its effectiveness. The majority of the patients (77%) had not consulted their physicians prior to utilization of CAM therapies. Herbal medicine was the most common form of CAM in depression, with a high satisfaction level. Thus, it is necessary to increase physicians' awareness in different fields of CAM.

## 1. Introduction

The prevalence of depression as the second most common psychological disorder has increased in developed and developing countries [1]. A previous study showed a twofold increase in the prevalence of depression in the United States between 1991 and 2002 [2]. Recent data have also shown that nearly 264 million people worldwide had depression [3]. Evidence has indicated that the prevalence of depression varied from 3% in Japan to 16.9% in the United States and 22.4% in Iran [1, 4]. Depression imposes human costs (disability and mortality) and financial strains on the society's economy. In Iran, these costs were estimated to be 420 thousand US dollars in 2009 and 210.5 billion US dollars in 2010 [5, 6]. Furthermore, depression could increase the risk of alcohol abuse and hospitalization as well as care costs [7]. Therefore, it is essential to pay more attention to all aspects of depression to promote its management.

Current treatments for depression emphasize medication use as well as nonpharmacological interventions. Several therapeutic groups (monoamino oxidases, tricyclic antidepressants, selective serotonin reuptake inhibitors, serotonin-norepinephrine reuptake inhibitors, and atypical antidepressants) have been used to treat depression [8]. However, the results of several large-scale meta-analyses have raised concerns regarding the efficacy and tolerability of commonly used selective serotonin reuptake inhibitors (SSRIs) [9, 10]. On the other hand, antidepressants have several side effects, such as dry mouth, dizziness, drowsiness, prolonged orgasms, decreased sexual function, constipation, inability to drive, headache, insomnia, and sadness [11, 12]. In addition, research has shown a link between antidepressants use and hepatic impairment and increased risk of diabetes [13, 14]. Therefore, the tendency to use nonpharmacological remedies for treating depression has increased. Nowadays, many nonpharmacological interventions, such as meaning therapy [15], cognitive group counseling [16], cognitive-behavioral therapy [17], cognitive rehabilitation [18], and Complementary Alternative Medicine (CAM) [19], aim at improving the treatment of depression.

In recent years, there has been an increasing interest in the use of CAM in the treatment of many patients [20]. Numerous studies have been conducted on the prevalence, type, and effectiveness of CAM. For instance, two meta-analyses conducted by Qin Xiang et al. showed that St John's wort and curcumin had significant clinical efficacy in ameliorating depressive symptoms [21, 22]. Xin Liu et al. conducted a study on 260 patients with depression and reported that 50% of the patients had used one of the complementary therapies in the past 12 months [23]. Another study carried out in the US indicated the use of complementary and alternative medicine among 40% of adults with moderate depression and anxiety [24]. Studies have revealed an increase in the prevalence of CAM use among people due to the side effects of drugs, nonsatisfaction with conventional therapies, physicians' communication skills impairments, and so forth [25]. Professional health providers also have different attitudes and practices regarding CAM, ranging from encouragement to ignorance and even opposition to CAM [26]. Some physicians believe that the use of CAM may lead to a delay in diagnostic and lunch therapy procedures. On the other hand, many patients do not inform their physicians about complementary therapies, which increases the risk of complications, such as drug interactions and errors in the interpretation of laboratory results [27].

Since depression is a chronic illness with a high economic and psychological burden on society, detailed information regarding CAM use can be valuable for health providers. However, little information is available regarding the use of CAM for the treatment of depression worldwide and there are no studies in this field in Iran. Thus, the present study aims to evaluate the use of CAM and its associated factors in depression.

## 2. Method

### 2.1. Design of the Study and Ethical Issues

This cross-sectional study was conducted on 300 depressed patients referred to the Yasuj Neurology and Psychiatric Clinic between 2019 and 2020. This study was approved by the local Ethics Committee of Shiraz University of Medical Sciences (IR.SUMS.REC.1399.316). Written informed consent forms were also obtained from all participants.

### 2.2. Sample Size and Study Population

Adults diagnosed with depression by a psychiatrist based on the DSM5 guidelines were eligible to enter the study. According to the previous studies conducted on the issue, a 300-subject sample size was determined for the study [28]. The participants were selected by the convenience sampling method.

### 2.3. Questionnaire and Data Collection

The study data were collected using a valid semistructured questionnaire that has previously been published elsewhere (28), which consisted of 29 open- and close-ended questions. The questionnaire consisted of four sections. The first section included demographic information, including age, gender, education level, occupation, ethnicity, duration of the disease, hospitalization and nonhospitalization, body mass index (BMI), place of residence, and income level (categorized as low, intermediate, and high based on the monthly income). The second part of the questionnaire focused on the use of CAM, including the duration of CAM usage, main reasons for consumption of CAM, and familiarity with the method. The third section was on the type of CAM (type of herbs) used, consultation with a physician prior to the use of CAM, and assessing visits to CAM providers. And the last was about the level of satisfaction based on a Likert scale. The questionnaire took 20 minutes to complete.

### 2.4. Statistics

Frequency, mean, standard deviation (SD), and percentage were used to describe the data. A chi-square test was used to assess the relationships between the variables among CAM users and nonusers. In addition, binary logistic regression was used to evaluate the association between the demographic variables and CAM use. Crude and adjusted odds ratios (OR) and their 95% confidence intervals (CI) were estimated as well. All analyses were performed using the SPSS software, version 23 (IBM Corporation, Armonk, NY), and *p* < 0.05 was considered to be statistically significant.

## 3. Results

### 3.1. Background Data

This study was conducted on 300 patients with depression, including 70 males and 230 females (77%). The mean age of the patients was 41.47 ± 12.2 years and the mean duration of the disease was 4.49 ± 4.88 years. A significant difference was found between the CAM users and nonusers regarding the duration of the diseases (*p*=0.045) and BMI (*p*=0.007). The demographic characteristics of the participants with respect to the use and nonuse of CAM have been displayed in Table 1. In addition, the results of logistic regression analysis of the use of CAM in patients are presented in Table 2. The results indicated that CAM use was significantly associated with female gender, duration of the disease (more than three years), overweight, obesity, and self-employment (*p*=0.039, *p*=0.028, *p*=0.029, *p*=0.048, and *p*=0.044, respectively).

### 3.2. Use of CAM in Depression

CAM was used by 113 patients (37.6%), including 20 males (17.7%) and 93 females (82.3%). According to the results presented in Table 3, the most frequently utilized types of CAM were herbal medicine (97.35%) and pray therapy (23.89%). Moreover, the most widely used herbs were borage (77%), chamomile (46.9%), and lavender (21.2%). Further details are presented in Table 4.

### 3.3. Reasons for Using CAM and Levels of Satisfaction

**Figure 1:**
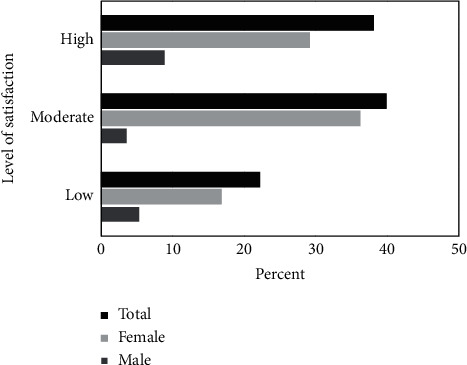
Level of satisfaction for CAM use depressive disorder.

Among the study participants, 71 CAM users (62.8%) reported that their main reason for using CAM was the effectiveness of this method (Table 5). The levels of satisfaction with CAM use have been depicted in Figure 1. Accordingly, 39.82% of the participants showed moderate and 38.05% showed high satisfaction levels.

### 3.4. Familiarity with CAM and Recommendation to Others

The majority of the participants (77%) had gotten familiar with CAM by their families and friends. Additionally, 81.4% of the patients had not been referred to CAM centers and 77% had not consulted their physicians prior to the utilization of CAM therapies. Furthermore, CAM users (80.5%) recommended CAM therapies to others. The details are presented in Table 6.

## 4. Discussion

This was the first study, which evaluated the prevalence of CAM use and its related factors in patients with depression in Iran. The results indicated that 37.6% of the patients had used one type of CAM to treat depression in the past year, with 77.78% showing moderate and high satisfaction levels. The most common type of CAM was herbal medicine. The results also revealed a significant relationship between CAM use and duration of the disease, female gender, obesity, overweight, and self-employment.

The use of CAM in the treatment of depression has been increasing worldwide [29]. The present study findings demonstrated that 37.6% of the patients had used one type of CAM to treat depression in the past year. The previous studies showed that the use of CAM among depressed patients varied from 17.8% to 54% [30]. Rhee et al. conducted a study in the US in 2017 and disclosed that 39.8% of the patients with severe depression had used CAM in the past year [24]. Wu P et al. also performed a study on women with depression and indicated that 54% of the patients had used CAM in the past year [29]. In another study published in Norway, 17.8% of the people with depression and anxiety had used CAM in the past year [31]. In another study conducted by the European Social Survey (ESS), the use of different methods of CAM in depression varied from 2.5% to 19.5% [27]. These differences in the prevalence of CAM usage might be attributed to cultural differences, socioeconomic status, gender, used questionnaires, the severity of the illness, and comorbidities.

Evidence has indicated that several types of CAM have been used concurrently with conventional medications for the treatment of depression [30, 32]. In the present study, the most common type of CAM was herbal medicine, which was in line with the findings of the previous studies [33, 34]. The most common medicinal plants used in this study were borage, chamomile, and lavender, which was consistent with the results of the study carried out by Jäger et al. in Denmark [35]. The results of a study conducted in Mexico also revealed that the most commonly used plant was asteraceae (chamomile belongs to the asteraceae family) [36]. A review study conducted by Pilkington et al. in Singapore showed that borage and lavender could affect the treatment of depression [37]. The use of medicinal plants might result from people's culture. The use of herbal medicines has a long history in Iran. Iranians have been using herbs as spices or home remedies for many years. Therefore, access to medicinal plants is easier and cheaper for Iranians. Yet, the disparity in the type of herbal medicines used in different countries might be associated with traditional health products, cultural contexts, socioenvironmental factors, and availability of herbal medicines.

Several studies have found that many factors, including gender, duration of the disease, chronicity, severity of the disease, and BMI, were associated with the use of CAM [27, 31, 38, 39]. The present study results showed that 77% of the participants were female, which was consistent with the findings of the previous studies, reporting that females were more likely to be depressed [23, 40, 41]. The present study findings also revealed a significant relationship between the duration of the disease (≥3) and the use of CAM, which was in agreement with the results obtained by Ahmad et al. [42]. In addition, a study by Demirci et al. demonstrated that the duration of the disease was statistically longer in CAM users (5.0 ± 5.9) than in nonusers [43]. In the current study, there was a significant relationship between BMI>25 kg/m^2^ and using CAM, which was in line with the results of the research by Blanck et al., which showed that 433 out of the 632 CAM users (68.5%) had BMI>25 kg/m^2^ [44]. In contrast, a previous study suggested a lower prevalence of use of several CAM modalities, such as relaxation, yoga, herbal medicine, massage, chiropractic, tai chi, and acupuncture, among obese adults compared to normal-weight individuals [45]. Education level and economic status could also influence the use of CAM. Previous studies have shown different relationships between the tendency to use CAM and education level. Some studies carried out in developing countries revealed a significant relationship between low education levels and CAM usage [46, 47], while other studies conducted in developed countries indicated a greater use of CAM among people with high education levels [27, 31, 38, 39]. Finally, some studies including the present one demonstrated a nonsignificant relationship between education level and the use of CAM. Yet, the majority of CAM users in the present study had diploma and below diploma degrees, which was consistent with the findings of the research performed by Farhoudi et al. and other studies conducted on the issue [28, 32, 48].

The previous studies revealed a significant relationship between high-income levels and the use of CAM in developed countries [27, 31, 38, 39]. However, the present study results showed no significant relationship between income level and the use of CAM. This discrepancy might be due to the type of population under investigation and classification of the socioeconomic status.

Generally, reasons for CAM usage and level of satisfaction could influence the use of CAM products in the community. Similar to the previous studies [49, 50], a great number of patients (62.8%) in the present study reported that their main reason for using CAM was the effectiveness of this method. Additionally, 77.8% of the patients showed moderate and high satisfaction levels with the use of CAM, which was higher compared to the previous studies. A systematic review demonstrated that the level of satisfaction with CAM use was 50.5% [34].

In the current study, the majority of the patients reported that their physicians were not aware of their use of CAM. In the same vein, the previous studies indicated that 95%, 89.9%, and 42% of the CAM users had not said anything about the use of CAM to their conventional physicians [28, 32, 49]. This unawareness could lead to dangerous herb-drug interactions. For example, borage might increase the bleeding risk among patients taking warfarin. In addition, the concomitant use of *Hypericum perforatum* and SSRI could lead to serotonin syndrome [51]. There are few academic training centers in the field of CAM in Iran, and most physicians have not received any training in this field. This might account for the ineffective relationships between physicians and patients in the field of CAM. Furthermore, friends and relatives were the most common sources of CAM recommendation, which was in agreement with the findings of other studies on the use of CAM [28, 52–54]. In the end, it should be noted that other conventional CAM methods in the world, such as yoga, acupuncture, and homeopathy, were less frequently used in the present investigation. This might have its roots in insufficient information about these methods among people or lack of reputable centers providing these services.

## 5. Conclusion

The prevalence of depression is high in developing and industrialized countries, and these patients tend to use CAM plus conventional treatments. In the present study, herbal medicines were the most common form of CAM used for the treatment of depression, with a high satisfaction level. Interestingly, most physicians were unaware of the use of CAM by patients. This might cause irreparable damages for patients who use CAM due to drug interactions. Therefore, it is necessary to educate patients about CAM and enhance physicians' awareness in different fields of CAM. Studies on the effects of various forms of CAM on depression and assessment of the effectiveness of CAM are also warranted.

## Figures and Tables

**Table 1:** Demographics of the participants with regard to use and nonuse of CAM in depression patients.

Associated factors	Total (*n* = 300)	Nonuser (*n* = 187)	User (*n* = 113)	*P* ^*∗*^ value
Gender				0.073
Male	70 (23.3)	50 (26.7)	20 (17.7)	
Female	230 (76.7)	137 (73.3)	93 (82.3)	

Age				0.112
≤35	107 (35.7)	70 (37.4)	37 (32.7)	
35 to 50	132 (44)	74 (39.6)	58 (51.3)	
>50	61 (20.3)	43 (23)	18 (15.9)	

Marital status				0.123
Married	259 (86.3)	157 (84.0)	102 (90.3)	
Single	41 (13.7)	30 (16.0)	11 (9.7)	

Educational status				0.469
Illiterate	63 (21)	43 (23.0)	20 (17.7)	
Diploma and below diploma	168 (56)	104 (55.6)	64 (56.6)	
Academic	69 (23)	40 (21.4)	29 (25.7)	

Job				0.055
Unemployment	212 (70.7)	128 (68.4)	84 (74.3)	
Employment	49 (16.3)	28 (15.0)	21 (18.6)	
Self-employment	39 (13)	31 (16.6)	8 (7.1)	

Income				0.377
Bellow intermediate	89 (29.7)	58 (31.0)	31 (27.4)	
Intermediate	154 (51.3)	98 (52.40	56 (49.6)	
Above intermediate	57 (19)	31 (16.6)	26 (23)	

Smoking				0.278
No	182 (60.7)	109 (58.3)	73 (64.6)	
Yes	118 (39.3)	78 (41.7)	40 (35.4)	

Alcohol use				0.329
No	290 (96.7)	179 (95.7)	111 (98.2)	
Yes	10 (3.3)	8 (4.3)	2 (1.8)	

Duration of disease (year)				0.045
≤3	163 (54.3)	110 (58.8)	53 (46.9)	
>3	137 (45.7)	77 (41.2)	60 (53.1)	

Hospitalization history				0.234
No	239 (79.7)	153 (81.8)	86 (76.1)	
Yes	61 (20.3)	34 (18.2)	27 (23.9)	

Residence				0.076
Urban	236 (78.7)	141 (75.4)	95 (84.1)	
Rural	64 (21.3)	46 (24.6)	18 (15.9)	

(BMI, body mass index)				0.007
Normal	107 (35.7)	79 (42.2)	28 (24.8)	
Overweight	112 (37.3)	65 (34.8)	47 (41.6)	
Obese	81 (27)	43 (23)	38 (33.6)	

^*∗*^ *P* value of chi-square test; CAM : Complementary Alternative Medicine.

**Table 2:** Sociodemographics correlation of CAM use.

Associated factors	Crude R 95% cl	Adjusted R 95% cl	*P* ^*∗*^ value
Gender			
Male	1	1	0.039
Female	1.69 (0.95, 3.04)	1.93 (1.03, 3.58)	

BMI			
Normal	1	1	
Overweight	2.04 (1.15, 3.61)	1.95 (1.08, 3.55)	0.028
Obese	2.49 (1.35, 4.60)	2.07 (1.08, 3.99)	0.029

Hospitalization history			
No	1	1	0.095
Yes	1.41 (0.79, 2.49)	1.72 (0.91, 3.25)	

Duration of disease, more than 3 years (year)			
No	1	1	0.048
Yes	1.62 (1.01, 2.59)	1.73 (1.005, 2.99)	

Age			
≤35	1	1	
35 to 50	1.48 (0.88, 2.51)	1.25 (0.69, 2.27)	0.462
>50	0.79 (0.40, 1.56)	0.55 (0.25, 1.20)	0.133

Job			
Unemployment	1	1	
Self-employment	0.39 (0.17, 0.89)	0.416 (0.18, 0.98)	0.044
Employment	1.14 (0.61, 2.14)	1.36 (0.70, 2.65)	0.362

^*∗*^Logistic regression; CAM : Complementary Alternative Medicine.

**Table 3:** Frequency of CAM usage in depression patients.

Type of CAM use	Total, *n* (%) 113 (100)	Male, *n* (%) 20 (17.7)	Female, *n* (%) 93 (82.3)
Acupuncture	2 (1.77)	0	2 (100)
Herb	110 (97.35)	20 (18.2)	90 (81.8)
Leech	2 (1.77)	1 (50)	1 (50)
Wet cupping	7 (5.26)	2 (28.6)	5 (71.4)
Yoga	1 (0.88)	0	1 (100)
Diet	2 (1.77)	0	2 (100)
Homeopathy	2 (1.77)	1 (50)	1 (50)
Pray	27 (23.89)	5 (18.5)	22 (81.5)
Dry cupping	1 (0.88)	0	1 (100)

CAM : Complementary Alternative Medicine.

**Table 4:** Frequency of type of herbs usage in depression patients.

Type of herbal use	Total, *n* (%) 113 (100)	Male, *n* (%) 20 (17.7)	Female, *n* (%) 93 (82.3)
Borage	87 (77)	15 (17.2)	72 (82.8)
Yellow chamomile	53 (46.9)	9 (17)	44 (83)
Lavender	24 (21.2)	4 (16.7)	20 (83.3)
Lemon balm	9 (8)	3 (33.3)	6 (66.7)
Valerian	15 (13.3)	5 (33.3)	10 (66.7)
Bitter orange blossom	16 (14.2)	2 (12.5)	14 (87.5)
Rose	10 (8.8)	0	10 (100)
Saffron	18 (15.9)	3 (16.7)	15 (83.3)
Cumin	12 (10.6)	4 (33.3)	8 (66.7)

**Table 5:** Reasons for CAM use in depression disorder patients.

Reasons for CAM use	Total, *n* (%) 113 (100)	Male, *n* (%) 20 (17.7)	Female, *n* (%) 93 (82.3)
Effectiveness	71 (62.8)	16 (22.5)	55 (77.5)
Accessibility	25 (22.1)	2 (8)	23 (92)
Harmless	8 (7.1)	1 (12.5)	7 (87.5)
Unhappy with conventional medical treatment	9 (8)	1 (11.1)	8 (88.9)

CAM : Complementary Alternative Medicine.

**Table 6:** Frequency of source of information, prescription by a physician, consultation, and recombination.

Variable	*N* (%) 113	Male *N* (%) 20	Female *N* (%) 93
Source of information			
Friends and family members	87 (77)	14 (16.1)	73 (83.9)
Physician	13 (11.5)	3 (23.1)	10 (76.9)
Media (TV, books, and magazines)	8 (7.1)	2 (25)	6 (75)
Herbalist and pharmacy	26 (23)	4 (15.4)	22 (75.6)

Prescription by physician			
Yes	21 (18.6)	5 (23.8)	16 (76.2)
No	92 (81.4)	15 (16.3)	77 (83.7)

Consultation before using by a physician			
Yes	26 (23)	5 (19.2)	21 (80.8)
No	87 (77)	15 (17.2)	72 (82.8)

Recommendation to others			
Yes	91 (80.5)	14 (15.4)	77 (84.6)
No	22 (19.5)	6 (27.3)	16 (72.7)

## Data Availability

The data supporting the findings of this study are available from the corresponding author upon request.
